# Supplementary material for: Lexical representation explains cortical entrainment during speech comprehension
Source: PLoS One. 2018 May 17;13(5):e0197304. doi: 10.1371/journal.pone.0197304 (PMC5957381; doi:10.1371/journal.pone.0197304)
Supplement: S1 Appendix — (PDF) [file pone.0197304.s002.pdf]

S1 Appendix Representing words as syntactic categories

Under the model's explanation of the Ding et al.'s results, similar outcomes should be obtained if vectors represent only the syntactic categories of the stimulus words. The question remains if this can be done without interpreting the stimuli beyond the individual words, because for many words the syntactic category (also known as part-of-speech; POS) is ambiguous until the word is understood in its sentence context.

As a first test of the feasibility of a 'POS only'-account, we replaced each word of the English [NP VP] stimuli by its most frequent POS in the ENCOW corpus (i.e., without considering the word's role in the sentence), yielding 13 different POS tags. Each was assigned a vector (available in S1 Data) such that all 13 vectors are orthogonal, that is, they identify POS without encoding any notion of similarity between syntactic categories. As much as possible without sacrificing orthogonality, vector values were randomly drawn from the original vectors (i.e., representing words), again for 12 simulated participants. The resulting frequency spectra (S2 Fig) again shows the power peaks at 1, 2, and 4 Hz. This suggests that it is possible that POS representations underlie the MEG findings. However, note that this result should not be taken as evidence for the cognitive or neural representation of syntactic categories (let alone for the particular set of POS tags in the ENCOW corpus) because similar outcomes were obtained using the original vector representations in which words only approximately cluster by POS.

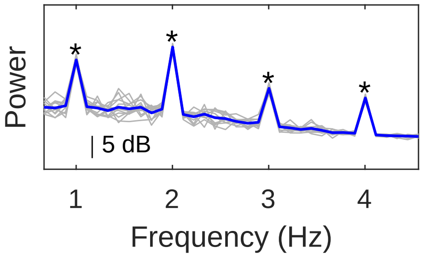

S2 Fig. Power spectra resulting from processing English [NP VP] sentences with each word replaced by its most frequent syntactic category.
