# Supplementary material for: Lexical representation explains cortical entrainment during speech comprehension
Source: PLoS One. 2018 May 17;13(5):e0197304. doi: 10.1371/journal.pone.0197304 (PMC5957381; doi:10.1371/journal.pone.0197304)
Supplement: S2 Appendix — (PDF) [file pone.0197304.s004.pdf]

S2 Appendix Vector lengths over the course of a sentence

Ding et al. presented participants with 90 Chinese sentences consisting of a 3- or 4-syllable NP followed by a 4- or 5-syllable VP. Overall MEG activity decreased strongly near the NP and sentence boundaries, which was taken as further evidence for neural tracking of phrasal and sentential structure.

We obtained vector representations for pinyin versions of the same stimuli (S2 Data) and took each vector's euclidean length as a measure for overall 'activation'. S3 Fig plots the average length as a function of syllable position in the sentence. There are indeed sharp drops after NP and sentence (VP) boundaries, corresponding to the human results. This is most likely caused by the fact that syllable strings that cross a phrase boundary rarely form a possible word beginning. Consequently, the first syllable of a phrase often starts a new cohort, and averaging over its many words yields a relatively short vector.

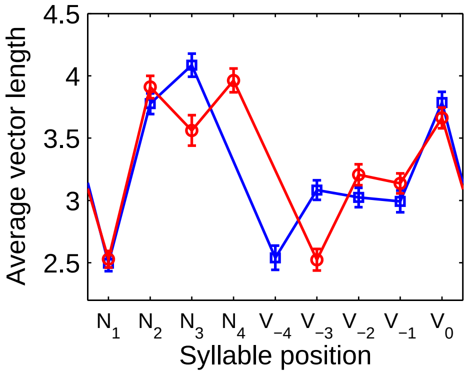

S3 Fig. Average euclidean length of vector representations at each point of 7- and 8-syllable Chinese [NP VP] sentences. Error bars indicate 95% confidence intervals. Plots are left aligned on the 3- or 4-syllable NP (labeled N_1 to N_4) as well as right aligned on the 4- or 5-syllable VP (labeled V_{-4} to V_0). Sentence with a 3-syllable NP have no N_4 and 4-syllable NP sentences have no V_{-4}.
