# Supplementary material for: Lexical representation explains cortical entrainment during speech comprehension
Source: PLoS One. 2018 May 17;13(5):e0197304. doi: 10.1371/journal.pone.0197304 (PMC5957381; doi:10.1371/journal.pone.0197304)
Supplement: S3 Appendix — (PDF) [file pone.0197304.s006.pdf]

S3 Appendix Localization of sentential- and phrasal-rate responses.

In an electrocorticography (ECoG) study using the English four-word [NP VP] sentences, Ding et al. identified the electrodes that selectively respond at the phrasal or sentential rate but not at the word-presentation rate. Across these electrodes, power at the phrasal and sentential frequencies were negatively correlated, which "demonstrates spatially dissociable neural tracking of the sentential and phrasal structures" (p. 162).

For each individual dimension of vector representations for the same stimuli sequences, we computed the peak size at each frequency as the power at that frequency minus the average over all frequencies within 0.5 Hz on either side. These peak size were then expressed as z-scores relative to the range from 0.5 to 4.5 Hz. Next, we identified vector dimensions that show a sentential- or phrasal-rate pattern but no word-rate pattern by selecting those with $z > 2$ at 1 or 2 Hz and $z < 1$ at 4 Hz. Across the 12 simulated subjects, 10.0% of vector dimensions met these criteria. On these dimensions, peak sizes at 1 and 2 Hz were negatively correlated ($r = -.43; p < .00001$) which shows that the lexical properties represented by the vectors are dissociated across vector dimensions in a way that corresponds to the the spatial dissociation between sentential- and phrasal-rate responses in Ding et al.'s ECoG experiment.
