# Supplementary material for: Lexical representation explains cortical entrainment during speech comprehension
Source: PLoS One. 2018 May 17;13(5):e0197304. doi: 10.1371/journal.pone.0197304 (PMC5957381; doi:10.1371/journal.pone.0197304)
Supplement: S4 Appendix — (PDF) [file pone.0197304.s007.pdf]

## S4 Appendix Setting model parameters

The model has three free parameters:

- $\mu$: The average arrival time of lexical information, in simulated milliseconds from word onset
- $\beta$: The width of the uniform distribution of arrival time of lexical information
- $\sigma$: The standard deviation of Gaussian noise

Appropriate values for these parameters were found by getting a new set of word vectors (with $N = 300$) for the English [NP VP] sentences. The objective was to find $\mu, \beta$, and $\sigma$ such that the frequency spectrum resembles the corresponding results from Ding et al. in this condition, that is, there are peaks at 1 Hz, 2 Hz, and 4 Hz, possibly a minor peak at 3 Hz, no other peaks, and a slight increase in power for lower frequencies.

S4 Fig shows the power spectra for each combination of $\mu \in \{25, 75, 125, 175, 225\}, \beta \in \{0, 25, 50, 75, 100\}$, and $\sigma \in \{0, .25, .5, .75, 1\}$. The three major peaks at 1 Hz, 2 Hz, and 4 Hz, and the minor peak at 3 Hz, are clearly visible for most combinations of parameter values. Only for very high levels of noise $\sigma$ or when both $\mu$ and $\beta$ are high (which means that much lexical information never arrives) are the power spectra mostly flat. These results motivated us to fix $\beta$ at 50 and perform a more fine-grained search through parameter space for low-to-medium levels of $\mu$ and $\sigma$. Based on visual inspection of the resulting power spectra in S5 Fig, we settled for $\mu = 40$ and $\sigma = 0.5$. The parameters are not over-fitted, as is clear from the fact that the same combination of values resulted in very similar power spectra using another set of vectors as well as on the Chinese sentences (Fig. 1).

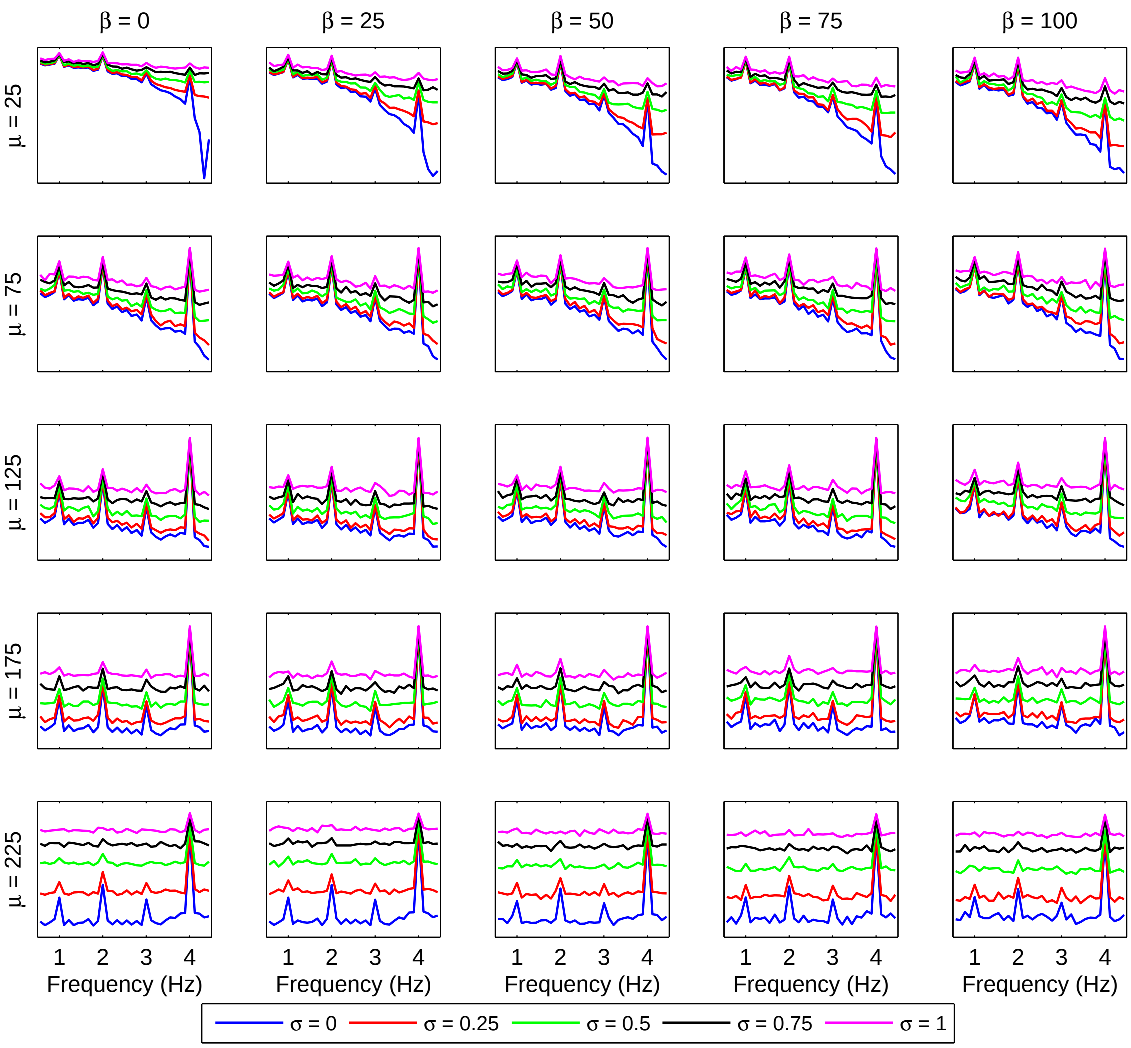


**S4 Fig. Power spectra resulting from processing English [NP VP] sentences, for different combinations of parameter values.**

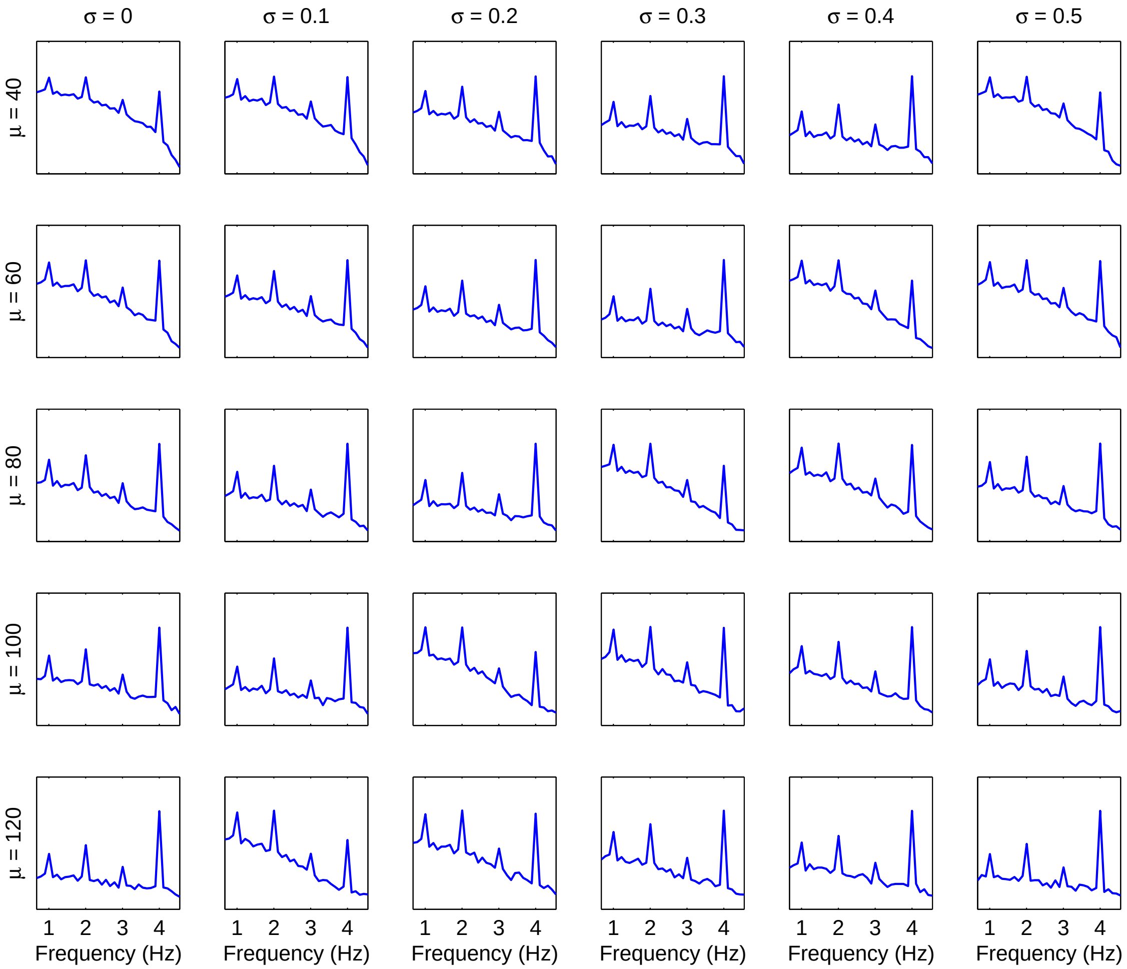


**S5 Fig. Power spectra resulting from processing English [NP VP] sentences, for $\beta = 50$ and different values of $\mu$ and $\sigma$.**
